# Coblator Arytenoidectomy in the Treatment of Bilateral Vocal Cord Paralysis

**DOI:** 10.1155/2015/487280

**Published:** 2015-09-17

**Authors:** Benjamin Googe, Andrew Nida, John Schweinfurth

**Affiliations:** ^1^University of Mississippi School of Medicine, University of Mississippi Medical Center, Jackson, MS 39216, USA; ^2^University of Mississippi Medical Center, Department of Otolaryngology, Jackson, MS 39216, USA

## Abstract

A 77-year-old female with bilateral vocal cord paralysis and dependent tracheostomy status after total thyroidectomy presented to clinic for evaluation of decannulation via arytenoidectomy. Preliminary data suggests coblation versus standard CO_2_ laser ablation in arytenoidectomy may provide benefits in terms of decreased tissue necrosis and patient outcome. The patient elected to proceed with arytenoidectomy by coblation. The initial procedure went well but postoperative bleeding required a return trip to the operating room for hemostasis. In the coming months the patient's tracheostomy tube was gradually downsized and eventually capped. She was decannulated eight months after surgery, speaking well and without complaints. Details of the surgical procedure and outcome will be discussed.

## 1. Introduction

Bilateral vocal fold immobility may result from trauma, disease, or idiopathic paralysis [1, 2]. Regardless of the etiology, the goal of therapy is the same, to establish a secure airway thereby permitting adequate airflow for respiration. Surgical management of bilateral vocal cord paralysis has evolved overtime, progressing from simple tracheotomy to vocal cordotomy and arytenoidectomy [3]. Carbon dioxide (CO_2_) laser ablation of the medial surface of the arytenoid and vocal fold has become standard of care in the management of persistent bilateral VCP [2, 4, 5]. Using coblation as a substitute for CO_2_ laser ablation may provide benefits in terms of decreased tissue site necrosis and improved patient recovery [6, 7]. Our patient suffered from bilateral vocal cord paralysis (VCP) due to a recurrent nerve injury from a total thyroidectomy. Here we present a case of bilateral VCP treated with coblator arytenoidectomy.

## 2. Materials and Methods

A retrospective analysis was performed on a 77-year-old female who presented to the senior author's (John Schweinfurth) clinic for airway evaluation. The patient had a total thyroidectomy one year before at an outside hospital and subsequently developed respiratory distress requiring tracheotomy secondary to bilateral VCP. She had done well in the past year with her tracheostomy tube but wished to explore further options for treatment of her VCP and possible decannulation. On exam, the patient was speaking well with Passy-Muir valve and denied dysphagia, choking, or dyspnea. Flexible bronchoscopy revealed fixation of the true vocal cords bilaterally in the midline with mild evidence of subglottic stenosis. Due to high preoperative body mass index (BMI) and tight glottis airway on exam, she was deemed a poor candidate for cordotomy alone. After discussing her options, the patient elected to proceed with surgery for left medial arytenoidectomy by coblation.

### 2.1. Surgical Technique

**Figure 1:**
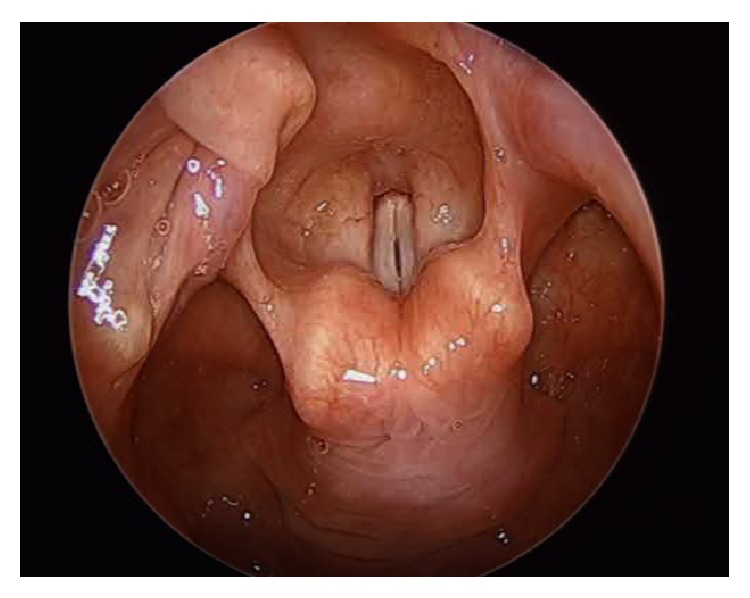
Intraoperative visualization of the larynx.

**Figure 2:**
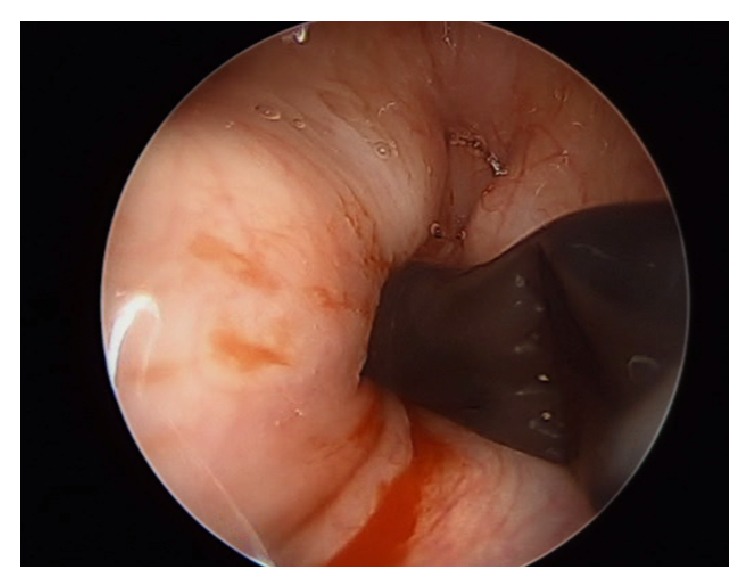
Laryngeal spasm following induction of coblation.

**Figure 3:**
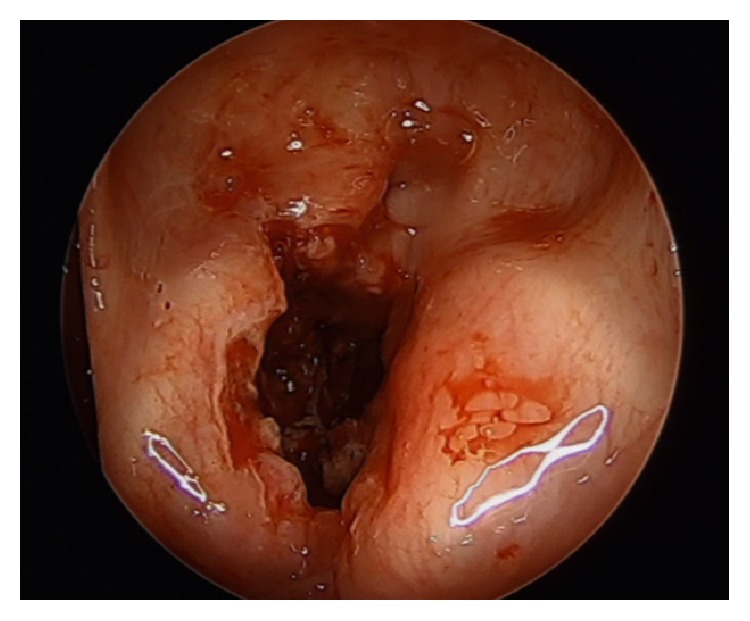
Final view of the larynx following completion of left medial arytenoidectomy.

The patient was taken to the operating room (OR), sedated with general anesthesia, and ventilated via her tracheostomy tube. A supraglottic laryngoscope was then inserted and 2 mL of 4% lidocaine was sprayed onto the glottis for topical anesthesia. The oral cavity, oropharynx, hypopharynx, and larynx were found to be normal with medialization of vocal folds bilaterally (Figure 1). Both cricoarytenoid joints moved normally on palpation. Afrin pledgets were applied to the left arytenoid. An ArthroCare ENT Coblator was utilized to resect soft tissue and cartilage of the left arytenoid beginning at the vocal process. The larynx was initially quite sensitive to the coblate function causing frequent spasms of the laryngeal musculature (Figure 2). As dissection proceeded deeper contraction seemed to subside but brisk bleeding was encountered. Hemostasis was attained using the coagulation function of the coblator. A sufficient amount of cartilage was removed to achieve adequate expansion of the airway (Figure 3). The patient was awakened from general anesthesia and transferred to the postanesthesia care unit in stable condition.

## 3. Results

In the two hours following surgery the patient began to expel bloody sputum and clots from her tracheostomy tube. Conservative management with Afrin and suctioning was unsuccessful and the patient was taken back to the OR for control of the laryngeal hemorrhage. A small area of bleeding was visualized on the anterior portion of the arytenoidectomy wound bed and was successfully cauterized with suction Bovie. She tolerated the procedure well and was discharged later that day without further complication.

The patient followed up monthly in the clinic after the procedure. Aside from mild edema visualized on laryngoscopy on the first visit, there were no complications. She denied complaints of bleeding, dysphagia, dyspnea, or choking and was speaking well with finger occlusion. Her tracheostomy tube was gradually downsized and intermittent capping was initiated on month six. She was decannulated eight months after surgery, speaking well and without complaints.

## 4. Discussion

While conservative therapies may be considered for unilateral VCP, cases of persistent bilateral VCP almost invariably require surgery for definitive treatment [2]. No matter what the procedure or technique is, the intrinsic goal of widening the airway remains the same, and the removal of obstructing tissue is required. Preliminary studies have shown coblator arytenoidectomy to be a viable option [8].

Coblation is emerging as a predominant modality in common otolaryngologic procedures including tonsillectomy and nasal turbinate reduction and may offer advantages to CO_2_ laser ablation in the treatment of bilateral VCP [6, 9]. Coblation utilizes radiofrequency energy in self-supplied saline medium to generate a* plasma* field to disrupt tissues at relatively low temperatures and thereby decrease thermal damage to surrounding tissue. Postoperative pain has been shown to be significantly reduced in patients who received coblation tonsillectomy versus laser tonsillectomy, which is consistent with the limited pain complaints of our patient status after coblator arytenoidectomy [10].

One issue that seemed to arise early in the coblator arytenoidectomy was laryngeal muscle spasm (Figure 2). Early in the operative course this seemed to intermittently impair visualization of the ablation site but resolved as we penetrated deeper into the soft tissue. Due to the smaller and more restricted operative site in comparison with tonsillectomy, injection of local anesthetics to the musculature may improve visualization during the procedure in future patients.

Although postoperative bleeding was encountered in our patient requiring a short same-day reoperation for hemostasis, the patient seemed to recover well and was still discharged on the evening of the initial procedure. The patient was decannulated eight months after the operation.

## 5. Conclusion

This case demonstrates the procedure and benefits of arytenoidectomy in the management of bilateral VCP and provides an alternative to traditional CO_2_ laser ablation. We believe that in the coming years coblator arytenoidectomy will become a viable option in the treatment of bilateral VCP. Future study in the use of coblation for arytenoidectomy will further assess its advantages in the treatment of bilateral VCP.
